# Higher Rates of Emergency Oral Health Care Presentations Among Indigenous Australians: A Comparative Public Health Study

**DOI:** 10.3390/ijerph23020251

**Published:** 2026-02-17

**Authors:** Neeraj Vyas, Simranjit Cheema, Rachel Scobie, Barbie Fusitu’a, Gary Low, Albert Yaacoub, Muhammad Irshad, Stephen Cox, Mafaz Ullah

**Affiliations:** 1 Discipline of Oral Surgery, Sydney Dental School, Faculty of Medicine and Health, University of Sydney, Sydney, NSW 2750, Australia; 2 Nepean Centre for Oral Health, Nepean Blue Mountains Local Health District, Kingswood, Sydney, NSW 2747, Australia; 3 Aboriginal Health Unit, Nepean Blue Mountains Local Health District, Corner of Glebe & Springfield Place, Penrith, Sydney, NSW 2750, Australia; 4 Research Operations, Nepean Blue Mountains Local Health District, Faculty of Medicine and Health, University of Sydney, Sydney, NSW 2750, Australia; 5 School of Nursing and Midwifery, Western Sydney University, Penrith, Sydney, NSW 2751, Australia; 6 Periodontics Department, Prince Abdullah bin Abdulaziz bin Musaed Specialized Dental Center, Ministry of Health, An Nasiriyah, Arar 73241, Saudi Arabia

**Keywords:** oral health, dental emergency, Indigenous Australian, health disparities, public dental services

## Abstract

**Highlights:**

**Public health relevance—How does this work relate to a public health issue?**

- Service and access: Indigenous Australians have more emergency dental visits, showing the need for timely, preventive, and culturally safe dental care.
- Health equity and system burden: Heavy use of emergency and surgical care increases hospital and system load, highlighting the need for targeted public health strategies.

**Public health significance—Why is this work of significance to public health?**

- This study highlights preventable disparities in emergency dental care among Indigenous Australians, showing inequities in access to timely and preventive oral health services.
- Findings can inform public health strategies and resource planning to reduce hospitalizations, improve oral health outcomes, and promote health equity.

**Public health implications—What are the key implications or messages for practitioners, policy makers and/or researchers in public health?**

- Indigenous Australians experience higher rates of emergency dental presentations and extractions, emphasizing the need for preventive care.
- Public health strategies should focus on addressing oral health inequities.

**Abstract:**

Background: This study compared the reasons for dental presentations between Indigenous and non-Indigenous Australian adults at the Nepean Centre for Oral Health, NSW, Australia. Methods: A retrospective cohort study was conducted on adult patients presenting between 1 July and 31 December 2019. Clinical notes were audited and categorized as either ‘emergency’ (pain, dental infection, dental trauma, loose teeth or dental implants, bleeding) or ‘conservative’ (dental check-ups, examinations, prosthodontic, restorative, periodontal, or non-emergency endodontic therapy). Results: A total of 4663 patients met the inclusion criteria; 61.6% were female, and 6.4% were identified as Indigenous Australians. Overall, 41.3% of presentations were for emergency dental care. Indigenous Australians were significantly more likely to present for dental emergencies compared with non-Indigenous (58.2% vs. 40.2%; χ^2^(1, N = 4663) = 53.4, *p* < 0.00001; OR = 2.07, 95% CI: 1.64–2.63), and were also more likely to undergo emergency tooth extractions (*p* < 0.05). Conclusions: Indigenous Australians demonstrated a higher proportion of emergency and oral surgery-related presentations and underwent tooth extractions as the primary treatment compared to their non-Indigenous counterparts. These findings suggest a higher burden of oral disease and delayed access to general dental services among Indigenous Australians, highlighting the need for targeted and culturally appropriate public health interventions.

## 1. Introduction

Indigenous Australians experience significantly poorer oral health compared to non-Indigenous Australians [1]. Since 1979, studies have consistently shown higher prevalence and severity of dental caries and periodontal disease among Indigenous Australians [2]. Indigenous Australians continue to experience both higher prevalence and severity of dental caries compared to non-Indigenous children [3]. Indigenous Australians also experience higher rates of potentially preventable hospitalizations (PPH) for oral health-related conditions, placing a substantial burden on the public health system [4,5].

Barriers to preventive dental care contribute to poorer oral health outcomes among Indigenous Australians [6]. Financial constraints, limited service availability, transportation difficulties, and concerns about culturally appropriate care often delay dental visits [7]. Indigenous Australians are frequently in some of the lowest socioeconomic groups in Australia [8]. Consequently, many access oral health services only when experiencing pain or dental emergencies, often requiring more invasive treatments and resulting in poorer oral health outcomes [9]. Although many Indigenous Australians are eligible for government-subsidized dental care, significant waiting times for treatment remain a concern, making regular dental visits difficult for a large population [10]. Similarly, access to oral health care facilities is limited in rural and remote regions, and this disproportionately affects Indigenous Australians, as a larger proportion of the Indigenous population lives outside major cities compared with non-Indigenous Australians [9].

Oral health literacy is closely linked to oral health outcomes. Lower oral health literacy has been consistently associated with poorer periodontal health and higher rates of dental caries [11]. Parker et al. reported that 83.9% of Indigenous Australians with low oral health literacy typically sought dental treatment only in dental emergency situations, which often resulted in tooth extraction compared to conservative treatment [12]. The lack of awareness of services that are available has also contributed to a lack of attendance, suggesting that fostering partnerships and communication is a requirement for addressing these concerns. A lack of awareness of available dental services further contributes to low attendance, suggesting the importance of dental education. Consequently, many Indigenous Australians access care only when experiencing pain or emergencies, frequently necessitating more invasive treatments and exacerbating oral health disparities.

Understanding the factors influencing dental attendance patterns is crucial for developing targeted interventions to improve oral health outcomes among Indigenous Australians. The existing literature indicates that Indigenous Australians are more likely than non-Indigenous Australians to seek oral health care for pain or acute conditions rather than for preventive care; however, the evidence remains limited and is largely based on survey study designs [13,14,15,16]. Furthermore, despite New South Wales (NSW) having the largest Indigenous population nationally, no published clinical studies have examined reasons for attendance at oral health facilities among Indigenous Australians in this state, representing a significant gap in the literature [13,14,15,16]. Therefore, the aim of this study is to identify the differences in reasons for attendance between Indigenous and non-Indigenous Australians at the Nepean Centre for Oral Health, NSW, Australia. It is hypothesized that Indigenous Australians are more likely to seek dental appointments for pain, emergencies, or problems rather than for check-ups or preventive services, compared to non-Indigenous Australians.

## 2. Materials and Methods

### 2.1. Ethical Approval

Ethical approval for this retrospective cohort study was obtained from both the Nepean Blue Mountains Local Health District Human Research Ethics Committee and the Aboriginal Health and Medical Research Council of New South Wales Human Research Ethics Committee (Approval No. 2022/ETH00758).

### 2.2. Patient Selection

This study analyzed all adult patients who presented for treatment at the Nepean Centre for Oral Health (NCOH) during the 6-month period between 1 July 2019 and 31 December 2019, prior to the COVID-19 pandemic. Patients were eligible for inclusion if they were aged 18 years or older and received an initial assessment or treatment provided by, or under the supervision of, a general dentist. Only initial appointments for a course of care within the study period were included to avoid duplication of patient records. Patients under 18 years of age and those who received treatment from dental specialists, postgraduate registrars, oral health therapists, dental hygienists, or dental prosthetists were excluded from this study.

Demographic information, including age, gender, and Indigenous status, was recorded alongside reasons for presentation and treatment provided. Emergency presentations included patients presenting with pain as a chief complaint, loose teeth or dental implants, dental trauma, uncontrolled bleeding or clinically diagnosed dental infections. Conservative presentation included patients seeking check-ups, comprehensive examinations, prosthodontic services, restorative treatments, periodontal therapy, or non-emergency endodontic therapy.

Emergency treatment was subclassified into: ‘dental extractions’, ‘pulp therapy’, ‘restorations’, ‘periodontal therapy’, ‘treatment declined’ or ‘other’. The ‘other’ category included ‘no treatment’, ‘denture adjustment’, ‘referral to minor oral surgery’, ‘referral to oral medicine’, ‘reappointment for treatment with subsequent failure to attend’, ‘management of temporomandibular disorder’, ‘referral to endodontist’, ‘incision and drainage’, ‘postoperative bleeding’, ‘splinting of teeth’, or ‘referrals to other clinicians. ‘Referrals to other clinicians’ included referrals to periodontics, prosthodontics, special needs dentistry, and plastic surgery, as well as oral health therapists.

Conservative treatment was subclassified into: ‘comprehensive examination’, ‘scale and clean’, ‘restoration’, ‘periodontal therapy’, ‘denture-related services’, ‘non-emergency tooth extraction’, ‘endodontic therapy’ or ‘other’. The ‘other’ category for conservative included no treatment, dental crowns, treatment for dentinal hypersensitivity, internal bleaching, treatment declined, occlusal splints, orthodontics, periodontal splint, radiographic examination, recontouring of restorations, review, referral to specialists (oral surgery, oral medicine, prosthodontist, endodontist, orthodontist) or referral to others (general medical practitioners, prosthetists, intravenous sedationists for dental treatment and oral health therapists).

### 2.3. Data Collection

To minimize bias in the interpretation of clinical records, two investigators (NV, SC) independently reviewed all included patients’ electronic records. Any discrepancies were resolved by a third investigator (MU). Data were extracted using an electronic dental record platform, Titanium patient management system developed by Titanium Solutions Limited (Albany, Auckland, New Zealand). Patient identifiers were removed to ensure confidentiality. Medical record numbers were coded and transferred to a master datasheet in Microsoft Excel^®^ Version 2021 (Microsoft Corporation, Redmond, WA, USA), assigning participant numbers from 1 to 8669. De-identified data related to outcomes were collected and prepared for analysis.

### 2.4. Statistical Analysis

Descriptive statistics were used to summarize patient demographics and presentation categories (categorical variables), reported as counts and percentages. Age was analyzed as a continuous variable and summarized using means and standard deviations. Group comparisons were performed using chi-square tests for categorical variables and independent sample *t*-tests for age. Univariable and multivariable binary logistic regression models were used to examine factors associated with emergency versus conservative presentation.

Software: statistical analyses were performed using IBM SPSS Statistics for Windows, Version 29 (IBM Corp., Armonk, NY, USA).

## 3. Results

### 3.1. Population Characteristics

**Figure 1:**
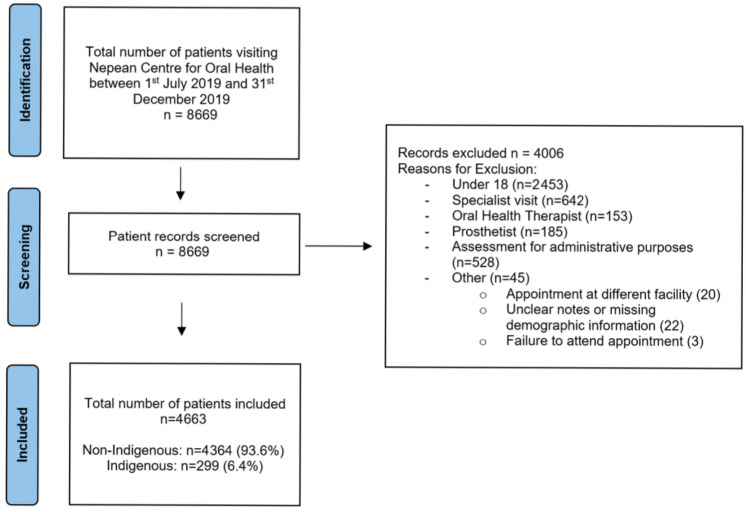
Selection of patients, according to inclusion and exclusion criteria.

A total of 8669 patients presented to the Nepean Centre for Oral Health (NCOH) between 1 July 2019 and 31 December 2019. After applying inclusion and exclusion criteria, 4663 patients were included in this study (Figure 1).

As shown in Table 1, Indigenous Australians comprised 6.4% of the total study population, including 291 Aboriginal individuals, five Torres Strait Islanders, and three who identified as both. Females predominated in the overall study population, particularly among Indigenous presentations, accounting for 61.6% and 70.9% of cases, respectively. Indigenous patients were also notably younger on average than non-Indigenous patients (40.2 ± 5.9 years vs. 56.0 ± 18.5 years).

#### 3.1.1. Presentation Types: Emergency vs. Conservative

Indigenous Australians were significantly more likely to present for emergency dental care than non-Indigenous patients (χ^2^(df = 1, N = 4663) = 53.4, *p* < 0.00001). Univariable logistic regression demonstrated that Indigenous status was associated with higher odds of emergency presentation (OR = 2.07; 95% CI: 1.64–2.63; *p* < 0.001) (Table 2).

#### 3.1.2. Types of Dental Emergencies

Pain was the common reason for emergency presentations, accounting for 87.7% of cases overall (Table 3), with a higher proportion observed among Indigenous (92.5%). Dental infections diagnosed as periapical abscesses, with or without draining sinuses, were recorded in 123 patients (6.4% of emergencies), with 54.5% presenting with facial swelling (Table 4). No statistically significant differences were found between Indigenous and non-Indigenous patients regarding the types of emergency presentations (*p* > 0.05), dental trauma, loose teeth or dental implants, and bleeding, likely due to the small number of cases in these categories.

#### 3.1.3. Treatments Provided for Emergency Presentations

Dental extractions were the most common treatment for emergency cases, accounting for 49.5% of all emergency treatments (Table 5). Indigenous patients were significantly more likely to undergo dental extractions compared with non-Indigenous patients (*p* < 0.05). No significant differences were observed between the groups for other treatment modalities.

#### 3.1.4. Conservative Dental Care

Conservative dental treatment was received by 59.8% of non-Indigenous patients and 41.8% of Indigenous patients, revealing a statistically significant disparity (*p* < 0.00001). Despite differences in the rate of conservative presentations, both groups received similar types of conservative care (Table 6).

#### 3.1.5. Influence of Gender and Age on Presentation Patterns

Females were more likely than males to seek emergency dental care (42.7% vs. 39.1%) (Table 7). Indigenous females demonstrated the highest proportion of emergency presentations (61.3%). Univariable logistic regression showed that female gender was associated with higher odds of emergency presentation (OR = 1.16; 95% CI: 1.03–1.31; *p* < 0.05) (Table 8). Increasing age was inversely associated with emergency presentations (OR = 0.97 per year increase; 95% CI: 0.97–0.97; *p* < 0.001) (Table 9). In multivariable analysis adjusting for age and gender, Indigenous status remained a significant predictor of emergency presentation (OR = 1.32; 95% CI: 1.03–1.69; *p* < 0.05) (Table 10).

## 4. Discussion

This study utilized a convenience sample comparing Indigenous and non-Indigenous adult Australians who attended the Nepean Centre for Oral Health at Nepean Blue Mountains Local Health District (NBMLHD) in Sydney for oral-related presentations. Indigenous Australians represent 3.6% of the NBMLHD population (*n* = 13,160), according to the 2016 report of the Australian Bureau of Statistics, which is slightly higher than the New South Wales average of 2.9% (*n* = 265,685) [17]. As such, the sample is not representative of the broader Indigenous adult population of New South Wales. While the findings provide valuable insights, they should be interpreted within the context in which the data were collected. The study population was drawn from a public oral health center where access is determined by specific eligibility criteria, which may limit the generalizability of the results to the wider community. In addition, the data were collected prior to the COVID-19 pandemic and therefore do not capture subsequent changes in health care access or health-seeking behaviors. The small size of the Indigenous population and the exclusion of socioeconomic variables, multiple geographic locations, comorbid conditions, and barriers to public health services are key limitations of this study. Despite these limitations, this study represents, to our knowledge, the only New South Wales-based clinical retrospective cohort study comparing the oral health-related presentations of Indigenous and non-Indigenous Australians in the adult population. Nevertheless, the use of routinely collected clinical data and the rigorous methodology employed enhance the internal validity and reliability of the findings.

The findings of this study, demonstrating that Indigenous Australians are significantly more likely than non-Indigenous Australians to seek emergency dental care rather than conservative or preventive services, and to undergo tooth extractions as part of emergency treatment, are consistent with the existing Australian literature. These patterns highlight the persistent disparities in oral health outcomes and in the nature of dental interventions received.

The literature consistently reports that the primary reason for dental attendance among Indigenous Australians is the presence of a problem or emergency, with proportions ranging from 54.4% to 77.1%. Kruger et al. [14], reporting on the largest sample of Indigenous patients, demonstrated the greatest disparity between emergency and non-emergency presentations, with 77.1% of Indigenous patients attending for emergency care compared with 22.9% for non-emergency care. In contrast, non-Indigenous patients attended for routine check-ups almost two and a half times more frequently (54.4%). Invasive surgical treatments occurred approximately twice as often in Aboriginal Medical Service clinics compared with public dental clinics, where restorative procedures were the most common treatments provided. National survey data from Brennan et al. [13] and Slade et al. [16] similarly reported that problem-based attendance accounted for 54.4% and 61.0% of dental visits, respectively, among Indigenous Australians. Comparable findings were reported in a survey by Arrow [15]. The findings of Brennan et al., Kruger et al., and Slade et al. were statistically significant. In contrast, Arrow’s study was limited by a small sample size, unclear demographic stratification, and a lack of statistical significance.

The findings of this study align with a growing body of global evidence demonstrating a disproportionate burden of dental disease among Indigenous populations. Across Australia, Canada, and New Zealand, Indigenous adults consistently experienced higher prevalence of untreated dental caries, greater tooth loss, and poorer self-rated oral health compared with non-Indigenous populations, with minimal heterogeneity observed between countries [18]. These patterns are similar to the findings of a recent systematic review and meta-analysis, which reported significantly higher caries prevalence and severity among Indigenous peoples worldwide, reflected in elevated DMFT scores across both permanent and deciduous dentitions and a greater burden of untreated decay [19]. Notably, the absence of meaningful differences in filled teeth observed in the present study is consistent with global evidence showing fewer restored teeth among Indigenous populations, suggesting reduced access to timely, preventive, and restorative dental care. Similar inequities in oral health outcomes and higher reliance on emergency dental services have been reported among Indigenous populations, suggesting that these disparities are not country-specific but represent a persistent global public health concern rooted in broader structural and systemic inequities [18,20,21].

A notable finding in the literature is the disproportionately high proportion of Indigenous females presenting for oral health care. In this study, Indigenous females accounted for 70.9% of all dental presentations and 61.3% of emergency dental visits, compared with Indigenous males. Although a similar trend was observed among non-Indigenous Australians, the gender disparity was smaller. The predominance of Indigenous females in seeking oral health services aligns with previous studies, which have reported proportions ranging from 62.1% to 66.7% [13,14,15,16].

The risk of dental infections requiring hospitalization is heightened by the increased prevalence of dental caries and periodontal disease, contributing to higher rates of emergency dental presentations among Indigenous Australians [20]. In 2023–24, the rate of potentially preventable hospitalizations due to dental conditions (per 1000 population) was higher for Indigenous Australians (5.6 per 1000 population) than for non-Indigenous Australians (3.3 per 1000 population). These rates of potentially preventable hospitalizations have increased over time, and with rates rising further with increasing geographic remoteness [22].

Although this study did not report on barriers to oral health services, financial constraints and limited-service availability associated with geographic remoteness have been widely reported as key barriers to accessing regular dental care among Indigenous Australians. National survey data from Slade et al. [16] and Brennan et al. [2] indicate that Indigenous Australians are more likely to delay or avoid dental care due to cost. Similarly, Kruger et al. reported the greatest disparity in emergency dental attendance, with emergency presentations accounting for 77.1% of appointments among Indigenous patients compared with 45.6% among non-Indigenous patients [14]. Notably, a large proportion of Indigenous participants from remote regions of Western Australia, where barriers related to cost and service availability are particularly pronounced. Consistent with these findings, the Australian Institute of Health and Welfare (AIHW) has identified cost as the most commonly reported barrier to accessing dental care in Australia [1].

Our findings in this study highlight an urgent need for further research and nationwide clinical data collection to identify disparities and barriers to oral health services for Indigenous Australians, which is essential for informing policy and closing gaps in attendance patterns. This study also provides a framework for collecting reliable data across Sydney and remote regions of Australia. Identifying the specific barriers faced by Indigenous Australians in accessing preventive dental care is crucial. Additionally, exploring the potential benefits of Aboriginal Community Controlled Health Services (ACCHSs) for dental care delivery may also be valuable [23].

## 5. Conclusions

This study highlights significant disparities in oral health service utilization between Indigenous and non-Indigenous Australians adults; Indigenous patients were more likely to present for emergency dental care and to undergo tooth extractions as the primary treatment, reflecting a reliance on problem-based rather than preventive care. These findings should be interpreted in light of this study’s limitations. Nevertheless, this study suggests the urgent need for targeted, culturally appropriate interventions to improve access to preventive dental services. Engaging with Indigenous communities and exploring the potential of Aboriginal Community Controlled Health Services may be key strategies for addressing these inequities and improving oral health outcomes. Future research should include larger Indigenous sample sizes and incorporate socioeconomic variables, multiple geographic locations, comorbid conditions, and barriers to accessing public dental services to provide a more comprehensive understanding of these disparities.

## Figures and Tables

**Table 1:** Patient demographic by gender and Indigenous status; total presentations for emergency and conservative appointments by Indigenous.

	Male	Female	Emergency	Conservative
	*n* (%)	*n* (%)	*n* (%)	*n* (%)
**All patients (*n* = 4663)**	1789 (38.4)	2874 (61.6)	1927 (41.3)	2736 (58.70)
**Indigenous** **(*n* = 299)**	87 (29.1)	212 (70.9)	174 (58.2)	125 (41.80)
**Non-Indigenous** **(*n* = 4364)**	1702 (39.0)	2662 (61.0)	1753 (40.2)	2611 (59.80)

**Table 2:** Binary logistic univariable regression model (Indigenous status).

Risk Factor	Beta	Standard Error	95% CI		Odds Ratio	95% CI	
			Lower	Upper		Lower	Upper
**Indigenous Status**	0.729	0.1212	0.492	0.967	2.073 *	1.635	2.629

Dependent variable: emergency and conservative. Intercept: −0.398 (standard error: 0.031). * Statistically significant (*p* < 0.05) (chi-square tests).

**Table 3:** Breakdown of the different types of emergency presentations.

Emergency	Pain	Dental Infection	Dental Trauma	Loose Tooth	Bleeding
	*n* (%)	*n* (%)	*n* (%)	*n* (%)	*n* (%)
**All patients (*n* = 1927)**	1690 (87.7)	123 (6.4)	18 (0.9)	92 (4.8)	4 (0.2)
**Non-Indigenous (*n* = 1753)**	1529 (87.2)	115 (6.6)	17 (1.0)	90 (5.1)	2 (0.1)
**Indigenous (*n* = 174)**	161 (92.5)	8 (4.6)	1 (0.6)	2 (1.1)	2 (1.1)

(*p* > 0.05). (Chi-square tests).

**Table 4:** Number of patients that presented with an infection and had facial swelling.

*p* > 0.05	Total Infection	Facial Swelling
	*n*	*n* (%)
**All patients**	123	67 (54.5)
**Non-Indigenous**	115	62 (53.9)
**Indigenous**	8	5 (62.5)

**Table 5:** Treatment delivered to patients presenting for an emergency appointment.

Emergency	Extraction	Pulp Therapy	Restoration	Periodontal Therapy	Treatment Declined	Other
	*n* (%)	*n* (%)	*n* (%)	*n* (%)	*n* (%)	*n* (%)
**Total Treatment** **(*n* = 1976)**	978 (49.5)	115 (5.8)	481 (24.3)	175 (8.9)	79 (4.0)	148 (7.5)
**Non-Indigenous** **(*n* = 1801)**	878 (48.8)	103 (5.7)	450 (25.0)	165 (9.2)	77 (4.3)	128 (7.1)
**Indigenous (*n* = 175)**	100 (57.1)	12 (6.9)	31 (17.7)	10 (5.7)	2 (1.1)	20 (11.4)

Dental extractions (*p* < 0.05). Others (*p* >0.05). (Chi-square tests).

**Table 6:** Treatment among patients presenting for conservative treatment.

Conservative (*n* = 2736)	Comprehensive Examination	Scale and Clean	Restoration	Periodontal Therapy	Denture Services	Extraction (No Pain)	Endodontic Therapy	Other
	*n* (%)	*n* (%)	*n* (%)	*n* (%)	*n* (%)	*n* (%)	*n* (%)	*n* (%)
**Total Treatment (*n* = 3238)**	1012 (31.3)	446 (13.8)	913 (28.2)	51 (1.6)	301 (9.3)	184 (5.7)	56 (1.7)	275 (8.5)
**Non-Indigenous** **(*n* = 3093)**	965 (31.2)	427 (13.8)	873 (28.2)	46 (1.5)	287 (9.3)	177 (5.7)	53 (1.7)	265 (8.6)
**Indigenous** **(*n* = 145)**	47 (32.4)	19 (13.1)	40 (27.6)	5 (3.4)	14 (9.7)	7 (4.8)	3 (2.1)	10 (6.9)

‘Other’ treatment included (275 total): crowns, dentinal hypersensitivity, internal bleaching, no treatment, treatment declined, occlusal splints, orthodontics, periodontal splint, radiographic examination only, recontouring of restorations, review, referral to specialists (oral surgery, oral medicine, prosthodontist, endodontist, orthodontist), referral to others (general practitioner, prosthetist, intravascular sedationist, oral health therapist), and presentation to acute care clinics for check-up. (*p* < 0.05). (Chi-square tests).

**Table 7:** Reason for attendance by gender demographics.

	Overall Emergency	Overall Conservative	Indigenous Emergency	Non-Indigenous Emergency	Indigenous Conservative	Non-Indigenous Conservative
Total	*n* (%)	*n* (%)	*n* (%)	*n* (%)	*n* (%)	*n* (%)
Male (*n* = 1789)	700 (39.1)	1089 (60.9)	44 (50.6)	656 (38.5%)	43 (49.4)	1046 (61.5)
Female (*n* = 2874)	1227 (42.7)	1647 (57.3)	130 (61.3)	1097 (41.2)	82 (38.7)	1565 (58.8)

**Table 8:** Binary logistic univariable regression model (gender).

Parameter	Beta	Standard Error	95% CI		Odds Ratio	95% CI	
			Lower	Upper		Lower	Upper
**Gender = Female**	0.148	0.0614	0.027	0.268	1.159 *	1.028	1.307
**Gender = Male**	0				1		

Dependent variable: emergency and conservative. Intercept: −0.442 (standard error: 0.048). * Statistically significant.

**Table 9:** Binary logistic univariable regression model (age).

Parameter	Beta	Standard Error	95% CI		Odds Ratio	95% CI	
			Lower	Upper		Lower	Upper
**Age**	−0.032	0.0017	−0.035	−0.029	0.968 *	0.965	0.972

Dependent variable: emergency and conservative. Intercept: 1.387 (standard error: 0.096). * Statistically significant.

**Table 10:** Binary logistic multivariable regression model.

Parameter	Beta	Standard Error	95% CI		Odds Ratio	95% CI	
			Lower	Upper		Lower	Upper
**Indigenous Status**	0.276	0.1272	0.027	0.526	1.318 *	1.027	1.691
**Gender = Female**	−0.036	0.0647	−0.163	0.090	0.964	0.849	1.095
**Age**	−0.031	0.0017	−0.035	−0.028	0.969 *	0.966	0.972

Dependent variable: emergency and conservative. Intercept: 1.360 (standard error: 0.1130). * Statistically significant.

## Data Availability

The raw data supporting the conclusions of this article will be made available by the authors upon request.
